# Sorption of Cu(II), Zn(II) and Pb(II) Ions in an Aqueous Solution on the PVC-Acetylacetone Composites

**DOI:** 10.3390/polym11030513

**Published:** 2019-03-18

**Authors:** Elzbieta Radzyminska-Lenarcik, Katarzyna Witt

**Affiliations:** Faculty of Chemical Technology and Engineering, UTP University of Sciences and Technology, PL 85326 Bydgoszcz, Poland; katarzyna.witt@utp.edu.pl

**Keywords:** PVC, acetylacetone, polymer composites, sorption, copper(II), zinc(II), lead(II)

## Abstract

The possibility of removing Cu(II), Zn(II) and Pb(II) ions by sorption on new PVC-based composite materials with different contents of acetylacetone (acac) and porophor was investigated. Composites were characterized using a scanning electron microscope and by infrared spectral analysis (FTIR). Sorption tests were conducted at 20 °C. It has been shown that the equilibrium is established in about 4 h. The reduction in ion concentration in the solution depended on the content of both acac and porophor in the composite. The maximal reduction in ion concentration ranged from 8% to 91%, 10–85% and 6–50% for Cu(II), Zn(II) and Pb(II) ions, respectively, depending on the composite composition. The best results were obtained for the composite containing 30% *w*/*w* of acac and 10% of porophor. For this composite, the sorption capacity after 4 h sorption for Zn(II), Cu(II) and Pb(II) ions was 26.65, 25.40, and 49.68 mg/g, respectively. Kinetic data were best fitted with a pseudo–second-order equation.

## 1. Introduction

Zinc, copper and lead are among the most important metals used in many areas of industry and economy of a given country (strategic metals) [1,2]. The still growing utilization and exploitation of these metals leads to an overall increase in their prices and stimulates a particular interest in even low-grade raw materials for their production. Hence, metal-bearing wastes are becoming more and more desirable raw materials [3,4].

The heavy metals from sewage could be a serious threat for the environment as well as for living organisms, because they are not biodegradable and tend to accumulate in living organisms [5]. Many of them are toxic (lead, mercury, cadmium, copper) or carcinogenic [6,7,8,9]. This is why metals should be removed [3,4,10]. Methods for recovering metals from industrial waste are gaining more and more significance [4,11,12].

For the last few decades, solvent extraction has been widely employed a technique for processing low-grade metalliferous raw materials [13]. This technique has been frequently used in the extraction of some non-ferrous metals [14,15,16,17,18]. An increasing demand for metal production has led to a search for more efficient and economical methods required by industry in terms of waste purification [19].

Many technologies, such as adsorption, precipitation, membrane filtration, and ion exchange, have been used to remove metal pollutants from water [20]. However, only adsorption has proven to be economical and efficient for removing heavy metals [21], organic pollutants [22] and dyes [23] from polluted waters.

The most commonly used adsorbent is activated carbon [21,24,25,26,27,28,29]. Due to the high costs of activated carbons (both production and regeneration) [30], cheap, available and renewable adsorbents are in demand [19,20,21,31,32,33].

Recently, an increasing interest in natural sorbents, e.g., chitosan [34,35,36,37,38,39,40], has been observed. Such sorbents are also waste byproducts from food and wood industries, as well as residues from the processing industry (including fruit and vegetable peelings, nut shells, seeds, straw, bark, and other forms of biomass) [19,41,42,43,44,45,46,47,48,49,50,51,52].

These materials are readily accessible and inexpensive. However, due to their variable composition, it is difficult to obtain replicable results. What is more, in relation to the metals being adsorbed, their selectivity is quite poor.

Hence, there is still a need for new, cheap, eco-friendly, effective and selective—sorption materials [10,11,52,53,54].

In our recent publications, we have demonstrated that the use of both acetylacetone (acac) [55] and its derivatives [56,57] as carriers in PVC-based polymer inclusion membranes allows for efficient and selective recovery of Zn(II) and Cu(II) ions from aqueous solutions [55,56,57] and galvanic wastes [58].

Currently, a significant increase of environmental pollution associated with the accumulation of the most harmful heavy metals, mainly: cadmium, lead, zinc copper, nickel and chromium, has been observed. These metals persist in the environment for a long time, which is why their concentration must be control and their excess should be removed.

The aim of the present work was to test the ability of PVC-acetylacetone composites to remove zinc, copper and lead ions from model solutions using the sorption method.

## 2. Materials and Methods

The sorption material used in the tests were PVC-based polymer composites promoted with acac. Their main components were the compounds listed below.

### 2.1. Polymer Composite Components

**Figure 1:**
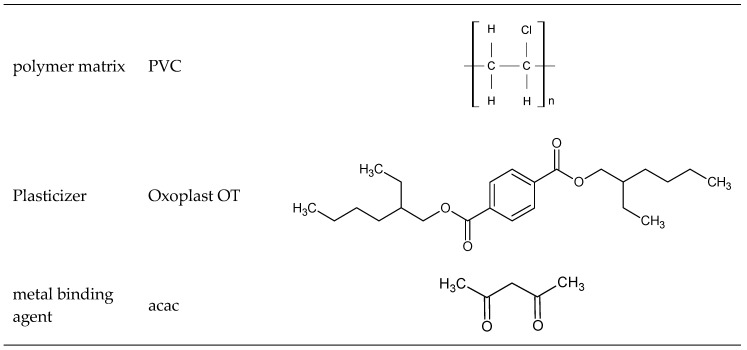
Main components of PVC-based polymer composites promoted with acac: polyvinyl chloride (PVC), bis(2-ethylhexyl) terephthalate (Oxoplast OT) and acetylacetone (acac).

On Figure 1 the main components of PVC-based polymer composites of are mentioned. Additionally in composites a PATSTAB 2301 was used as a stabilizer, and porophor Expancel 930 DUX 120 together with sodium chloride (NaCl) were used as sorption surface enhancing substances.

### 2.2. Composite Preparation

The process for preparing polymer composites is described in the patent application P.425353 [59]. A two-step preparation procedure was used to produce composites. The blend was produced in a Z-blade mixer at 105 °C and at a rotational speed of 60 min^−1^. To this end, suspension grade PVC (ANWIL Company, Wloclawek, Poland) and a thermal stabilizer (Promodent Invest Chemicals, London, UK) were introduced into the mixer chamber. The content was then mixed for 5 min. To a pre-heated PVC-stabilizer mixture, a mixture of liquid ingredients in a narrow stream was added for about 1 min, namely a mixture obtained by mechanical mixing of a plasticizer (Grupa Azoty Company, Kedzierzyn-Kozle, Poland) with acetylacetone (acac) (Avantor Performance Materials Poland Company, Gliwice, Poland) for 5 min at 23 °C. The mixture was stirred under the same conditions until PVC grains absorbed the liquid ingredients, eventually obtaining a dry blend (about 15 min). Subsequently, the mixture was cooled to room temperature (23 °C). In case of composites D and E, at this stage, Expancel 930 DUX 120 porophor (Boud Minerals Company, Lincolnshire, UK) was additionally introduced into the mixture and mixed with a mechanical stirrer (at rotational speed of 1200 min^−1^) for 5 min. To the obtained mixture, in case of composite E, sodium chloride (Avantor Performance Materials Poland Company, Gliwice, Poland) grinded by a blade mill to a dust form (particle size of about 50 µM) was introduced using a high-speed stirrer. The content was stirred for 10 min at rotational speed of 1200 min^−1^. The thus obtained blends were extruded using a single-screw extruder. The processing temperature was as follows: charging hopper—18 °C, zone I—60 °C, zone II—120 °C, zone III—130 °C, head—135 °C. Extrusion was carried out through circular cross-section dies having 3 mm in diameter and 40 mm in length. Afterwards, the mixture was cooled in air and grinded with a granulator. 

In Table 1, exact amounts of components used in the preparation of polymer composites are presented.

From the obtained composite E, sodium chloride was washed out by shaking in distilled water. This salt was used particularly as an agent for increasing the specific surface of the material, since its washing out from the active material gives the composite with an irregular, jagged and porous structure.

### 2.3. Sorption Process

To study the sorption process of heavy metals, each time 1 g ± 0.0001 g of the obtained composite materials (A–E) were weighed. Heavy metal solutions were prepared from nitrates (Zn (NO_3_)_2_·6H_2_O, Cu (NO_3_)_2_·3H_2_O and Pb (NO_3_)_2_, all from Avantor Performance Materials Poland S.A. (formerly POCH S.A.), Gliwice, Poland). For each metal ion, its (initial concentration) analytical concentration was 0.01 mol/dm^3^. Stock metal ion solutions were adjusted with ammonia (Avantor Performance Materials Poland S.A., Gliwice, Poland) to pH 8.0 (pH-meter MeterLab PHM240, Radiometer, Copenhagen, Denmark). For sorption testing, 50 cm^3^ of prepared stock solutions were used. The time of mixing for each composite (A–E) with metal salt solutions was: 0.5 h, 1 h, 2 h, 4 h, 8 h, 12 h and 24 h. Tests were performed at the temperature of 20 °C and at the atmospheric pressure.

## 3. Results and Discussion

### 3.1. FTIR Analysis

**Figure 2:**
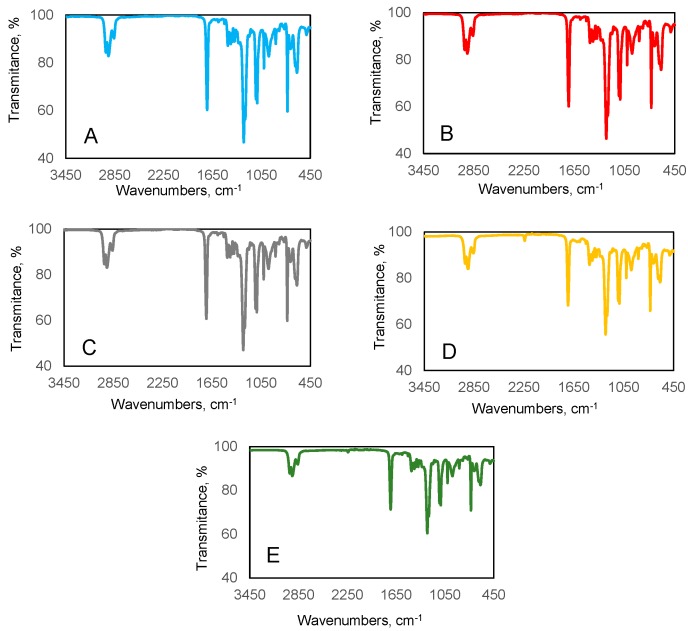
ATR-FTIR spectra of the tested polymer composites.

FTIR spectra of tested polymer composites were measured with a Bruker ALPHA Spectrometer at a wavenumber range of 450–4000 cm^−1^. ATR-FTIR spectra of the studied composites are shown in Figure 2.

No significant changes are observed between spectra of particular composites. The interpretation of infrared spectra was made using IRPal 2.0 software. Table 2 shows indicated bonds of characteristic bands which were found on ATR-FTIR spectra.

Figure 2 and Table 2 show that tested composites have similar chemical composition, but between this components do not exist any new stable chemical bonds.

### 3.2. SEM Analysis

**Figure 3:**
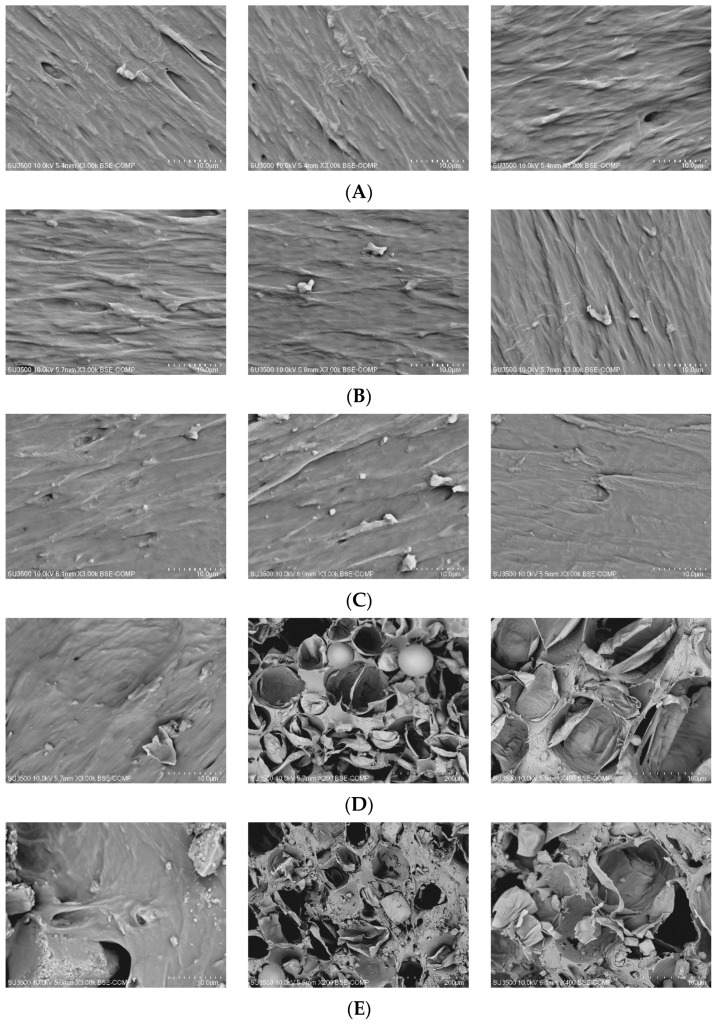
SEM images of the test polymer composites.

Scanning electron microscopy (SEM) (Hitachi SU3500 SEM/EDS (Energy-Dispersive Spectroscopy), Hitachi, Tokyo, Japan) was used to characterize the polymer composite surfaces. The obtained images are shown in Figure 3.

The surfaces of D and E composites are significantly more diverse than A, B, C composites. Surfaces of A, B, C composites have a very compact structure without visible pores. Presence of additional substances e.g., blowing agent in D and E composites cause huge changes in the surface structure. Moreover, on the images of composite E sodium chloride crystals are clearly noticeable. After rinsing the salt from the surface of composites a roughness structure is formed, which causes an increased active surface of the composite.

### 3.3. Sorption Process

Table 3, Table 4 and Table 5 show the concentration of Zn(II), Cu(II) and Pb(II) ions after the sorption at different times ranging from 0.5 h to 24 h on composite materials with various acac content (10% *w*/*w* in composite B and 30% *w*/*w* in composites A, D, E) and porophor content (10% *w*/*w* in composite D and 5% *w*/*w* in composite E) compared to composite C which contained neither acac nor porophor.

Results, which are presented in Table 3, Table 4 and Table 5, indicate that acac-free composite C does not bind any of the tested metal ions. Thus, the sorption of these cations from the solution determines formation of chelate complexes with acac contained in the composite.

As is known, acac forms—stable complexes with many d-electron metals. This ability is illustrated by the following Equations (1).

Therefore, the sorption efficiency of acac-containing composites is greater when compared to the same sorbents without this component.

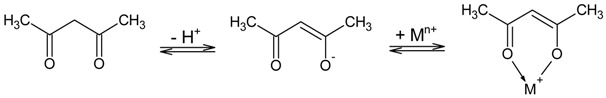
(1)

The amount of metal ion, which are binded in complex compound depends on stability constants of this complexes with acac.

The values of the logarithms stability constant of Zn(II), Cu(II) and Pb(II) complexes with acac are 5.05, 8.25 and 4.57, respectively [60].

The amount of metal ions adsorbed by 1 g of sorbent (*q_t_*) was calculated from Equation (2):

(2) $$q_{t}=\frac{\left(c_{0}-c_{t}\right)\cdot V}{m}$$

where *q_t_*—sorption capacity [mg/g], *V*—volume of the solution [dm^3^], *m*—mass of the sorbent [*g*].

The values of the sorption capacity of the tested composites after 4 h of sorption are presented in Table 6.

**Figure 4:**
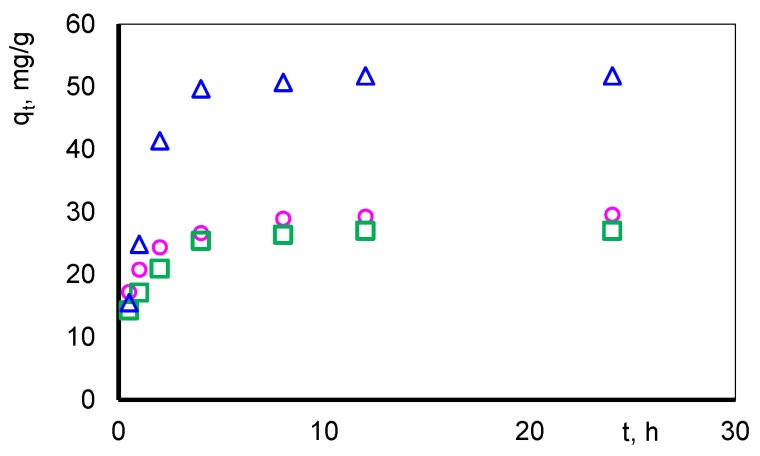
Sorption capacity of composite D vs. time for Zn(II) (o), Cu(II) (□) and Pb(II) (∆) ions.

Figure 4 presents dependence of sorption capacity vs. time for the most effectively sorbent (composite D).

In first stage of the sorption process a rapid increase of sorption capacity is observed (*qt*), which is related to the large number of available active places in relation to the amount of sorbed complexes. Tested complexes are quickly sorbed on the surface of sorbent. As the process progresses, their quantity gradually decreases and *qt* reaches a constant value. The equilibrium level is set after 240 min.

The regeneration of the composites was evaluated with 0.5 mol/dm^3^ HCl. The sorbent is stable for several sorption-desorption cycles.

**Figure 5:**
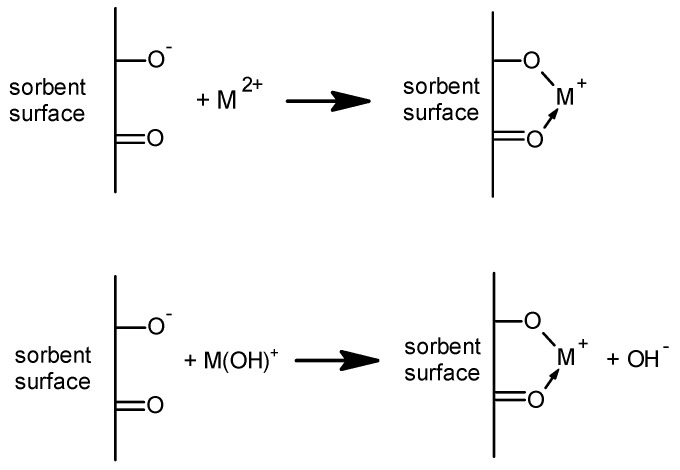
The sorption mechanism of metal ions on PVC-acac composites.

The proposed sorption mechanism of metal ions on PVC-acac composites is given in Figure 5.

### 3.4. Equilibrium Study

As the Boyd and Reichenberg equations [61,62] for the kinetic data analysis are suitable for spherical sorbents in the presented paper the pseudo-first-order (PFO) Equation (3) and pseudo-second-order kinetic models (PSO) Equation (4) were applied.

(3) $$log\left(q_{e}-q_{t}\right)=log\;q_{e}-\frac{k_{1}}{2.303}\cdot t$$

(4) $$\frac{1}{q_{t}}=\frac{1}{k_{2}\cdot q_{e}^{2}}+\frac{1}{q_{e}}\cdot t$$

where *q_e_*—experimental values of sorption capacity [mg/g], *k_1_*—equilibrium rate constant of pseudo-first-order adsorption [min^−1^], *k_2_*—pseudo-second-order rate constant of adsorption [mg/g·min^−1^].

Comparing the calculated kinetic parameters for pseudo-first-order (PFO-order) and pseudo second-order (PSO-order) reaction, due to the linear relationship *t/qt* vs. *t* and good agreement with experimental data (R^2^ ≈ 1) it was shown that the PSO-order kinetic model is fully suitable for describing the sorption process. 

**Figure 6:**
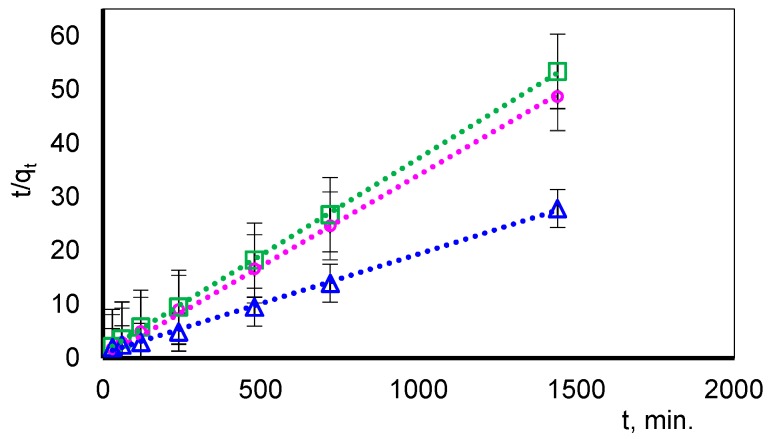
The PSO function matching to the experimental data obtained in the adsorption of Zn(II) (o), Cu(II) (□) and Pb(II) (∆) ions on composite D.

Linear plots of *t/q_t_* versus *t* are shown in Figure 6. The data obtained with correlation coefficients (R^2^) of Zn(II), Cu(II) and Pb(II)-composite D were 0.998, 0.998 and 0.993, respectively. The calculated q_2_ value estimated from the pseudo-second-order kinetic model is very close to the experimental values (*q_e_*). These results suggested that the studied adsorption systems followed the pseudo-second-order kinetic model.

The obtained data are presented in Table 7.

### 3.5. Metal Recovery

Concentrations of metals in the solution after a specified sorption time were analyzed by atomic absorption spectroscopy (AAS Spectrometer, Solar 939, Unicam, UK).

The percentage of metal ion removal (R) from the solution was calculated using the following equation:

(5) $$R=\frac{\left(c_{0}-c_{t}\right)}{c_{0}}\cdot 100\%$$

where *c_t_* is the metal ion concentration at a given time (mol/dm^3^), and *c_0_* is the analytical metal ion concentration (mol/dm^3^).

**Figure 7:**
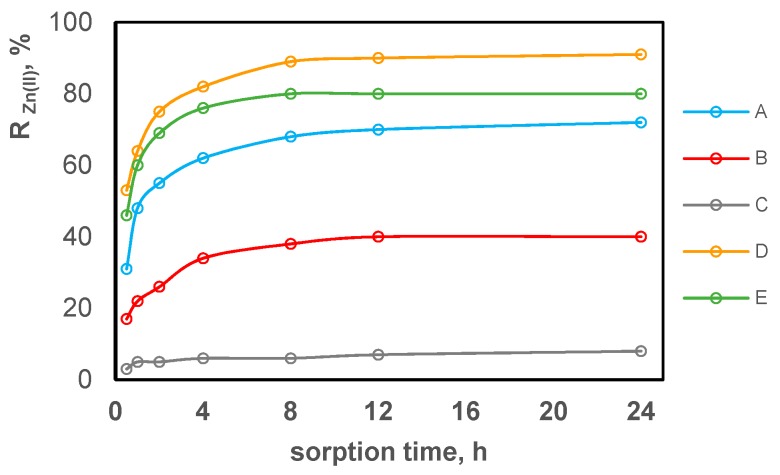
The reduction in zinc ion concentration expressed as a percentage of sorption on PVC-based composites promoted with acac depending on the contact duration.

**Figure 8:**
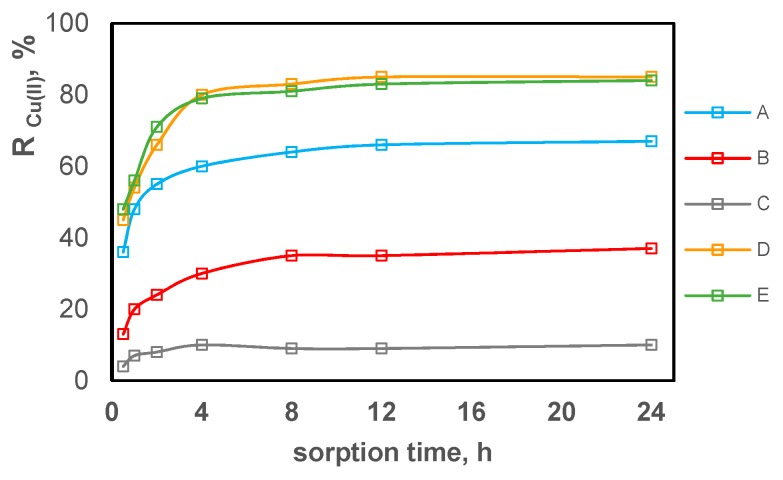
The reduction in copper ion concentration expressed as a percentage of sorption on PVC-based composites promoted with acac depending on the contact duration.

**Figure 9:**
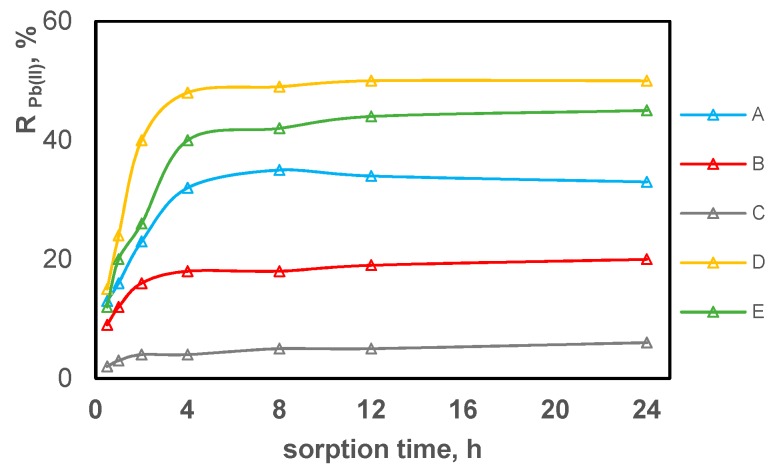
The reduction in lead ion concentration expressed as a percentage of sorption on PVC-based composites promoted with acac depending on the contact duration.

Using the Equation (5), the concentration reduction for each metal ion on each test composite (A–E) was calculated. The results are shown in Figure 7, Figure 8 and Figure 9 separately for each tested metal ion in relation to the sorption time.

By comparing the results shown in Figure 7, Figure 8 and Figure 9, it can be concluded that the sorption process occurs on all test composite materials and its efficiency depends on the composite composition. The equilibrium is reached in about 4 h, after which the ion concentration in the solution is practically unchanged. Zn(II) ions are sorbed most effectively, while Pb(II) ions are sorbed the least effectively. In terms of the efficiency of Zn(II), Cu(II) and Pb(II) sorption, the test composite materials can be ordered as follows C > B > A > E > D. The sorption efficiency increases with the acac content in the composite. Composite B containing 10% *w*/*w* of acac presents only slightly higher sorption of tested metal ions compared to composite C which contains no acac in its composition. Composites containing 30% *w*/*w* of acac (composites A, D, E) show the most effective reduction in the concentration for all tested metal ions.

**Figure 10:**
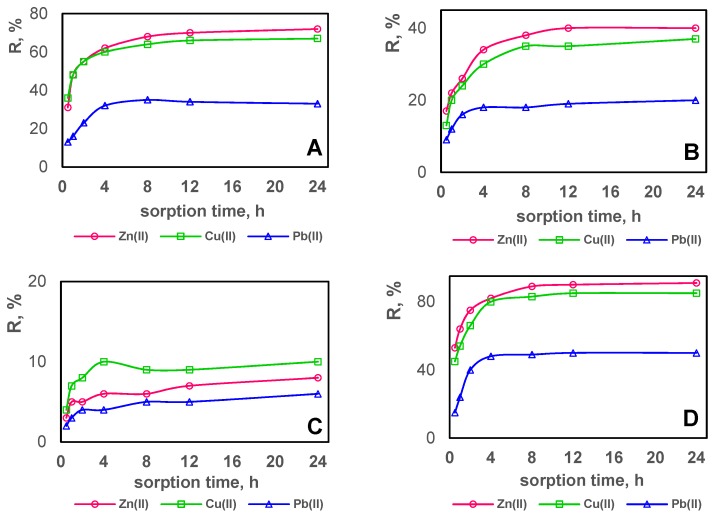

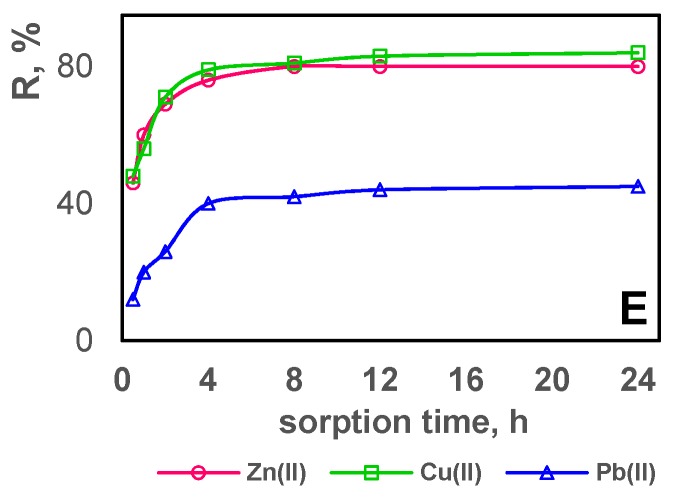
The reduction in Zn(II), Cu(II) and Pb(II) ion concentration expressed as a percentage of sorption on PVC-based composites (**A**–**E**) depending on the reagent contact duration.

The sorption efficiency of the obtained composite materials was compared by analyzing the relation between the reduction in Zn(II), Cu(II) and Pb(II) ion concentration and the time of sorption on all composites (Figure 10).

The highest concentration reduction for all metal ions was obtained using composite D. After the 24-h sorption, the reduction of Zn(II) ion concentration was 91%, 80%, 72%, 40% and 8% for composite D, E, A, B, C, respectively. For composites D, E, A, B, C, the reduction of Cu(II) and Zn(II) ions decreased in series D = E > A > B > C in the case of Cu(II) ions and D > E > A > B > C for Pb(II) ions, and amounted to a maximum of 84%–85% for Cu(II) (composites E and D) and 50% for Pb(II) (composite D). 

However, this efficiency may be further improved by increasing the composite surface by both the addition of the porophor itself (composite D), as well as the addition of sodium chloride and porophor mixture (composite E). 

### 3.6. Comparison of the Results with the Literature Data

The obtained results were compared with the given literature data concerning biosorption on activated carbons from plant waste and other sorbents (zeolite acrylamide and biomass) (Table 8).

From comparison of data, which were summarized in Table 8, shows that the new PVC-acac composite (composite D) have higher sorption efficiency against zinc(II) ions than activated carbons (obtained from: walnut shells, apricot stone, almond pits, pistachio shell) and natural zeolites. The composite is just as effective against zinc(II) and copper(II) ions as the acrylamide composite, but is less effective against lead(II) ions.

## 4. Conclusions

The sorption process of Zn(II), Cu(II) and Pb(II) ions does occur on PVC-based composite promoted with acac and its efficiency depends on the composite composition and on the additives which increase the sorption surface. The equilibrium is reached after about 4 h. 

Zn(II) ions are sorbed most effectively, while Pb(II) ions are sorbed least effectively. The sorption efficiency increases with the acac content in the composite. Composites containing 30% *w*/*w* of acac (composites A, D, E) show the most effective reduction in the concentration for all tested metal ions. This efficiency may be further improved by increasing the composite surface by the addition of the porophor itself (composite D), as well as the addition of sodium chloride and porophor mixture (composite E). 

The highest reduction in the concentration of all metal ions in the solution was observed for PVC-acac-porophor composite sorbent (composite D). After the 24-h sorption, the reduction in Zn(II), Cu(II) and Pb(II) ion concentration was 91%, 84% and 50%, respectively. Kinetic data were best fitted with pseudo–second-order equation.

Composites may contain PVC recovered from wastes. 

## Figures and Tables

**Table 1:** Amounts of components used in the preparation of polymer composites.

Component	Polymer Composite, Parts by Weight				
	A	B	C	D	E
PVC Neralit 601	100	100	100	100	100
PATSTAB 2301	4	4	4	4	4
Oxoplast OT	50	50	50	50	50
acac	30	10	0	30	30
Expancel 930 DUX 120	-	-	-	10	5
NaCl	-	-	-	-	100

**Table 2:** Indicated bonds in polymer composites.

Maximum Value of Wavenumber of Characteristic Band, cm^−1^	Indicated Bonds
614	C–H, C–Br
731	C–H, S–OR, NH
874	C–H, S–OR, NH
959	P–H, P–OR, =NOH, N–O
1019	C–F, P–H, P–OR, Si–OR, C–O
1102	C–F, C–O, C–N, C=S, P–H, P=O, Si–OR, C=O
1267	C–F, Ar–N, –CH_3_, P=O, N–O C–O, C–H
1463	–CH_2_–, –CH_3_, Ar C–C
1717	C=O
2243	C=C, CN, Si–H
2909	CH, OH (COOH)
2922	CH, OH (COOH), –CH_2_–
2929	CH, OH (COOH), –CH_2_–

**Table 3:** Sorption time-dependent concentration of Zn(II) ions after the sorption on PVC-based composites promoted with acac. Initial concentration of Zn(II) ions C_0_ = 0.01 mol/dm^3^.

Sorption Time, h	C_Zn(II)_, mol/dm^3^				
	A	B	C	D	E
0.5	0.0069	0.0083	0.0097	0.0047	0.0054
1	0.0052	0.0078	0.0095	0.0036	0.0040
2	0.0045	0.0074	0.0095	0.0025	0.0031
4	0.0038	0.0066	0.0094	0.0018	0.0024
8	0.0032	0.0062	0.0094	0.0011	0.0020
12	0.0030	0.0060	0.0093	0.0010	0.0020
24	0.0028	0.0060	0.0092	0.0009	0.0020

**Table 4:** Sorption time-dependent concentration of Cu(II) ions after the sorption on PVC-based composites promoted with acac. Initial concentration of Cu(II) ions C_0_ = 0.01 mol/dm^3^.

Sorption Time, h	C_Cu(II)_, mol/dm^3^				
	A	B	C	D	E
0.5	0.0064	0.0087	0.0096	0.0055	0.0052
1	0.0052	0.0080	0.0093	0.0046	0.0044
2	0.0045	0.0076	0.0092	0.0034	0.0029
4	0.0040	0.0070	0.0090	0.0020	0.0021
8	0.0036	0.0065	0.0091	0.0017	0.0019
12	0.0034	0.0065	0.0091	0.0015	0.0017
24	0.0033	0.0063	0.0090	0.0015	0.0016

**Table 5:** Sorption time-dependent concentration of Pb(II) ions after the sorption on PVC-based composites promoted with acac. Initial concentration of Pb(II) ions C_0_ = 0.01 mol/dm^3^.

Sorption Time, h	C_Pb(II)_, mol/dm^3^				
	A	B	C	D	E
0.5	0.0087	0.0091	0.0098	0.0085	0.0088
1	0.0084	0.0088	0.0097	0.0076	0.0080
2	0.0077	0.0084	0.0096	0.0060	0.0074
4	0.0068	0.0082	0.0096	0.0052	0.0060
8	0.0065	0.0082	0.0095	0.0051	0.0058
12	0.0066	0.0081	0.0095	0.0050	0.0056
24	0.0067	0.0080	0.0094	0.0050	0.0055

**Table 6:** The sorption capacity of PVC-acac composites after 4 h of sorption.

Metal Ion	Sorption Capacity, mg/g				
	Composite				
	A	B	C	D	E
Zn(II)	20.15	11.05	1.95	26.65	24.70
Cu(II)	19.05	9.53	3.18	25.40	25.08
Pb(II)	33.12	18.63	4.14	49.68	41.40

**Table 7:** Parameters of the kinetic models for the adsorption of Zn(II), Cu(II), Pb(II) ions on composite D (*c*_0_ = 0.01 mol/dm^3^, *m* = 1 g, *V* = 50 cm^3^, *t* = 0.5–24 h, *T* = 293 K).

Metal Ions	q_e_	PFO			PSO		
		q_1_	k_1_	R^2^	q_2_	k_2_	R^2^
Zn(II)	29.58	7.86	0.001	0.721	29.61	0.034	0.0998
Cu(II)	26.99	4.18	0.004	0.854	27.02	0.037	0.0998
Pb(II)	51.75	12.63	0.012	0.906	51.77	0.091	0.993

**Table 8:** Comparison of sorption of Zn(II), Cu(II), Pb(II) ions on composite D with literature data.

Sorbent	Sorption Efficiency, %			Ref.
	Zn(II)	Cu(II)	Pb(II)	
**activated carbon from:**				[46]
walnut shells	71.0	97.5	96.2	
apricot stone	58.8	92.9	96.9	
almond pits	63.4	99.8	80.1	
pistachio shell	63.4	83	52.7	
**composite D**	91	85	50	[this work]
	**sorption capacity**, mg/g			
**composite D**	26.65	25.40	49.68	[this work]
Zeolite, clinoptilolite	0.5	1.64	1.6	[63]
Clay (polymethoxyethyl) acrylamide	20.6	29.8	81.02	[64]
torrefied poplar-biomass	-	-	30.00	[54]
